# Psychometric Testing of the Greek Version of the Clinical Learning Environment-Teacher (CLES+T)

**DOI:** 10.5539/gjhs.v8n5p59

**Published:** 2015-08-31

**Authors:** Evridiki Papastavrou, Maria Dimitriadou, Haritini Tsangari

**Affiliations:** 1 School of Health Sciences, Department of Nursing, Cyprus University of Technology, Cyprus; 2 School of Business, Department of Economics and Finance, University of Nicosia, Cyprus

**Keywords:** clinical learning environment, nurse education, psychometric testing, validation

## Abstract

Clinical practice is an important part of nursing education, and robust instruments are required to evaluate the effectiveness of the hospital setting as a learning environment. The study aim is the psychometric test of the Clinical Learning Environment+Teacher (CLES+T) scale-Greek version.: 463 students practicing in acute care hospitals participated in the study. The reliability of the instrument was estimated with Cronbach’s alpha coefficients. The construct validity was evaluated using exploratory factor analysis (EFA) with Varimax rotation. Convergent validity was examined by measuring the bivariate correlations between the scale/subscales. Content, validity and semantic equivalence were examined through reviews by a panel of experts. The total scale showed high internal consistency (α=0.95). EFA was identical to the original scale, had eigen values larger than one and explained a total of 67.4% of the variance. The factor with the highest eigen value and the largest percentage of variance explained was “supervisory relationship”, with an original eigenvalue of 13.1 (6.8 after Varimax rotation) and an explanation of around 38% of the variance (or 20% after rotation). Convergent validity was examined by measuring the bivariate correlations between the scale and a question that measured the general satisfaction. The Greek version of the CLES+T is a valid and reliable instrument that can be used to examine students’ perceptions of the clinical learning environment.

## 1. Introduction

Nurse education is an integral part of any healthcare delivery system as it has a major role to play in the educational development of graduates who can deliver safe, good quality nursing care. Nursing professionals’ knowledge, attitudes and skills are acquired both through formal education in institutions and through experience in the clinical area (Gaberson & Oermann, 2010). The clinical area represents an environment where the student nurse interact emotionally, physically and cognitively with their surroundings and fulfill their learning outcomes. Under these circumstances the clinical area is referred to as the ‘clinical learning environment’. Clinical learning environment (CLE) is viewed as an invaluable resource in familiarizing students with the reality of their professional role (Henderson, Cooke, Creedy, & Walker, 2012). Students being exposed to a range of clinical experiences (Edwards, Smith, Courtney, Finlayson, & Chapman, 2004) helps to broaden and deepen both cognitive and psychomotor skills (Souza, Venkatesaperumal, & Radhakrishnan, 2013), develop caring relationships and aid nurses in their professional evolution, as embodied within the attitudes of the nursing workforce (Haugan, Sørensen, & Hanssen, 2012).

Interest in the concept of clinical education as a determinant of quality nursing has gained increasing attention since 1980, leading to various nursing education reforms (Pollard, Ellis, Stringer, & Cockayne, 2007). Historically, a number of researchers investigated the effectiveness of the clinical learning environment (CLE) from students’ perspectives, employed quantitative (Saarikoski, Marrow, Abreu, Riklikiene, & Özbicakçi, 2007; Melender, Jonsén, & Hilli, 2013; Papathanasiou, Tsaras, & Sarafis, 2014; Dimitriadou, Papastavrou, Efstathiou & Theodorou, 2015), qualitative (Papp, Markkanen, & Von Bonsdorff, 2003; Mattila, Pitkäjärvi, & Eriksson, 2010) and mix methodology (Bisholt, Ohlsson, Engström, Johansson, & Gustafsson, 2014; Ip & Chan, 2005). The pedagogical atmosphere characterized by respect, acceptance and opportunities for learning with the mentor and clinical teacher alike have a stake in making clinical learning successful and reliable (Papp et al., 2003; Saarikoski et al., 2007; Dimitriadou et al., 2015). Also, student satisfaction was consistently identified as the most reliable index of a “good” clinical learning environment (Chan, 2001; Papastavrou, Lambrinou, Tsangari, Saarikoski, & Leino-Kilpi, 2010; Dimitriadou et al., 2015). For that reason, a variety of instruments for evaluating CLE from students’ perspective have been developed such as the CLEI-Clinical Learning Environment Inventory (Chan, 2001); the CLESDI-Clinical Learning Environment Diagnostic Inventory scale (Hosoda, 2006); the BES-CPE (Levett-Jones, 2009); the SECEE - Student Evaluation of the Clinical Education Environment (Sand-Jecklin, 2009); and the CLES - Clinical Learning Environment and Supervision Scale. The CLES is a 27-item scale (Saarikoski & Leino-Kilpi, 2002) developed to measure both the clinical learning environment and clinical supervision. Later, this scale was reviewed by the developers and a new sub-dimension added culminating in a 34-item CLES + T scale (Saarikoski, Isoaho, Warne, & Leino-Kilpi, 2008) and assesses 5 factors. They are as follows- pedagogical atmosphere, leadership style of the ward manager, supervisory relationship, the premises in the ward, and the role of the nurse teacher. The CLES+T instrument was used mainly and extensively in Europe in an effort to develop a powerful, multilingual tool for evaluating the quality of clinical learning. To date, the CLES+T scale has been translated into nine different languages, i.e., English, Finnish, Italian, Greek, Swedish, Dutch, Norwegian, German and Spanish. It has been demonstrated to be a valid and reliable tool among different international samples (Saarikoski et al., 2008; Johansson et al., 2010; De Witte, Labeau, & De Keyser, 2011; Henriksen, Normann, & Skaalvik, 2012; Tomietto et al., 2012; Bergjan & Hertel 2013; Saarikoski et al., 2013; Watson et al., 2014; Vizcaya-Moreno, Pérez-Cañaveras, De Juan, & Saarikoski, 2015). Since the quality of clinical learning depends on how well the practice curriculum is structured, the use of a reliable tool is helpful in obtaining information regarding the effective organization of clinical practice and the quality of it’s supervision. With this in mind, CLES+T is selected to test the psychometric properties with the aim to enhance cross-cultural collaboration.

## 2. Methods

### 2.1 Aim

The current study aim was to test the psychometric properties of the Greek version of the Clinical Learning Environment Scale + Teacher (CLES+T-GR) of Greek Cypriot students.

### 2.2 Participants and Settings

The target population of the study included all nursing students enrolled at the three Universities offering a Bachelor’s degree in Nursing within Cyprus. Only students who were practicing in hospitals were recruited, and those who were practicing in primary health care centers and other community care settings were excluded. 664 students fulfilled the inclusion criteria and were given the questionnaire. 463 questionnaires were returned, giving a response rate of 70.33%.

### 2.3 Ethical Considerations and Data Collection

The research proposal was submitted to the National Bioethics Committee, which is responsible for all research projects in Cyprus according to the Law (3558/2001). The permission for access to the field research was obtained by the Chairs of Nursing from each university. The aims of and the rationale for the study, and assurances that the data would be processed anonymously were included in the information letter supplied with the questionnaire. The questionnaires were given to the students individually immediately after they had completed their clinical placement, during a nursing laboratory lesson. After completion, each questionnaire was returned in a closed envelope. The completion of the questionnaire was considered as an informed consent for participation in the study.

### 2.4 Research Instrument

As mentioned the CLES+T scale consists of 34 items classified into 5 subscales: (1) pedagogical atmosphere on the ward; (2) supervisory relationship; (3) leadership style of the ward manager; (4) premises of ward nursing; and (5) role of the nurse teacher in clinical practice (Saarikoski et al., 2008). Respondents are asked to score their perception to each item on a 5-point Likert-type scale ranging from “very dissatisfied” to “very satisfied”. Also demographic data, hospital and ward type, length of clinical placement, number of meeting with the nurse teacher, motivational level on clinical placement, and level of satisfaction were collected.

### 2.5 Data Analysis

For demographic data and scale items, descriptive statistics (frequencies, percentages, means and standard deviations), skewness and kurtosis were used. The internal consistency of the Greek version of the instrument and each dimension was estimated with Cronbach’s alpha coefficients. Also, item analysis was conducted on the data, providing item-to-total correlations and Cronbach’s alpha if the item was deleted from the scale. Construct validity was evaluated using exploratory factor analysis (EFA) with Varimax rotation. Principal components analysis (PCA) was implemented as the extraction method in EFA. Convergent validity was examined by measuring the bivariate correlations between the scale/subscales and the question about the general satisfaction of nurses. Content, validity and semantic equivalence were examined through review by a panel of experts of the content of each item, its wording and the meaning of the items, after translation, in the context of the Cypriot culture (Squires et al., 2013).

## 3. Results

### 3.1 Sample Characteristics

The final sample included 463 participants. Among those, 38.7% were males and 61.3% females, with ages ranging from 18 to 34 years, a mean of 21.08 years and standard deviation 2.23 years. 149 participants studied in private universities, and 314 in the single public university.

### 3.2 The Individual Scale Items

Descriptive statistics (mean and standard deviation) for individual items were calculated. In order to examine the variability of the answers and test for significant deviations from normality, item skewness and kurtosis were also reported. Acceptable values of skewness and kurtosis, based on George and Mallery (2001), are those between -1.5 and +1.5, whereas values between -1 and +1 are considered excellent. The highest mean was of an item on the supervisory relationship subscale (item 18 in the scale - My mentor showed a positive attitude…), with a mean of 4.3, and a (low) standard deviation of 1.00. This item had marginal values of skewness and kurtosis as well. This verified that most of the answers were “agree” or “fully agree”, as opposed to negative attitudes, but the values were within the acceptable range. Although, in most of the items, there was a weak trend towards the positive attitudes (agree and strongly agree), there were no critical values (high positive or negative) of kurtosis or skewness for any item with all values being in the acceptable range. Therefore, transformations were not deemed necessary and all items were included in the analysis. These results are presented in Table 1.

**Table 1:** Descriptives, Internal consistency and Reliability (sequence as presented in the questionnaire)

Item	Mean	Std. Deviation	Skewness	Kurtosis	Corrected Item-Total Correlation	Cronbach Alpha if item Deleted
**Pedagogical Atmosphere,** 9 items, Cronbach’s alpha 0.875						

The staff were easy to approach	3.7646	1.06635	-.843	.183	.522	.948
I felt comfortable going to the ward at the start of my shift	3.0606	1.22013	-.159	-.893	.385	.950
During staff meetings(e.g. before shifts) I felt comfortable taking part in the discussions	4.0475	1.03296	-1.160	1.093	.493	.948
There was a positive atmosphere on the ward	3.8013	1.04210	-.840	.426	.620	.948
The staffs were generally interested in student supervision	3.2505	1.19427	-.248	-.733	.605	.948
The staff learned to know the students by their personal names	2.4514	1.31973	.500	-.870	.403	.950
There were sufficient meaningful learning situations on the ward	3.4190	1.08981	-.356	-.525	.620	.948
The learning situations were multi-dimensional in terms of content	3.3826	1.08331	-.289	-.449	.613	.948
The ward can be regarded as a good learning environment	3.6609	1.09773	-.508	-.476	.613	.948

**Leadership style in ward management,** 4 items, Cronbach’s alpha 0.849						

The WM regarded the staff on his/her ward as a key resource person	3.9391	1.03774	-.970	.620	.546	.948
The WM was a team member	3.5502	1.18464	-.628	-.351	.490	.949
Feedback from the WM could easy be consider a learning situation	3.3275	1.13437	-.323	-.525	.584	.948
The effort on individual employee was appreciated	3.3348	1.09000	-.286	-.425	.531	.948

**Nursing care,** 4 items, Cronbach’s alpha 0.812						

The ward nursing philosophy was clearly defined	3.6811	.98259	-.569	.090	.576	.948
Patients received individual nursing care	3.8304	1.02769	-.696	-.099	.512	.948
There were no problem in the information flow related to patients’ care	3.7609	1.01158	-.636	-.015	.471	.949
Documentation of nursing (e.g. nursing plans, daily recording of nursing procedures etc.) was clear	3.8824	.99743	-.730	.129	.589	.948

**Supervisory Relationship,** 8 items, Cronbach’s alpha 0.849						

My supervisor showed a positive attitude towards supervision	4.2673	1.05593	-1.482	1.504	.658	.947
I felt that I received individual supervision	3.7733	1.18752	-.734	-.326	.647	.947
I continuously received feedback from supervisor	3.9399	1.16111	-.857	-.219	.683	.947
Overall I am satisfied with the supervision I received	3.9690	1.22435	-1.057	.110	.705	.947
The supervision was based on a relationship of equality and promoted my learning	3.9498	1.15256	-.921	-.048	.703	.947
There was a mutual interaction in the supervisory relationship	3.9569	1.14764	-.987	.251	.714	.947
Mutual respect and approval prevailed in the supervisory relationship	4.0597	1.11053	-1.098	.456	.681	.947
The supervisory relationship was characterized by a sense of trust	4.0072	1.14320	-1.001	.173	.703	.947

**Role of nurse teacher,** 9 items, Cronbach’s alpha 0.937						

In my opinion, the NT was capable of integrating theoretical knowledge and everyday practice of nursing	3.8366	1.18799	-.834	-.179	.607	.948
The NT was capable of operational sing the learning goals of this placement	3.8293	1.18870	-.855	-.107	.601	.948
The NT helped me to reduce the theory-practice gap	3.6800	1.22331	-.704	-.389	.543	.948
The NT was like a member of the nursing team	3.4181	1.27918	-.417	-.804	.501	.949
The NT was able to give his or her expertise to the clinical team	3.5730	1.19551	-.561	-.455	.550	.948
The NT and the clinical team worked in supporting my learning	3.4900	1.22629	-.442	-.718	.573	.948
The common meetings between myself mentor and NT were comfortable experience	3.8491	1.15296	-.794	-.174	.594	.948
In our common meetings I felt that we are colleagues	3.8604	1.14160	-.776	-.232	.632	.947
Focus on meetings was in my learning needs	3.5643	1.25568	-.553	-.699	.552	.948

### 3.3 Internal Consistency and Reliability-Item Analysis

Cronbach’s alpha was used for testing the reliability of the 34-item scale, as well as for the five subscales, where values close to one are considered satisfactory. Also, item analysis was conducted to provide information about how well each individual item correlated to other items in the sub-scale where corrected item-to-total correlations below 0.30 are usually considered unacceptably low (Polit & Beck, 2011). Also, all of the inter-item correlations were examined with the reliability of each item being considered by finding if the it’s Cronbach’s alpha was deleted.

The results showed high internal consistency for the total scale (α=0.95). Similarly, the reliability of each sub-category was found to be high, ranging from 0.81 (“nursing care”) to 0.96 (“supervisory relationship”). The latter results are presented in table 1. Item analysis showed that when any item was deleted from the scale, the alpha was slightly lower or approximately the same as compared to when all the items were included, suggesting that deleting any item does not change the overall reliability significantly, and as such, that all the items contribute to the high reliability of the scale. A slight increase was seen in items “I felt comfortable going to the ward at the start of my shift” and “the staff learned how to know the students by their names”, however small (from 0.949 to 0.950). The items can thus be considered reliable.

Corrected item-to-scale correlations varied from 0.38 to 0.71, showing that all correlations were satisfactory, that is, above 0.3. Finally, out of more than 250 inter-item correlations between the 34 items (not reported), only two correlations exceeded 0.80 (in the supervisory relationship factor). Thus, in general, the results suggest that within the scale no items duplicated each other. The corrected item-to-scale correlations and Cronbach’s alpha if the item was deleted appear in Table 1.

### 3.4 Content Validity and Semantic Equivalence

The Greek Version of the CLES-GR was translated and back-translated following a specific step procedure (Papastavrou et al., 2010) after obtaining consent from the authors. Although the content validity of the Greek version of the CLES-GR has been established, the questions were reviewed by five experts as the questionnaire was modified by the designers and re-named to include the nurse teacher. The expert panel agreed that the CLES+T-GR reflected the situation in the clinical practice environment, i.e., that the items were suitable and relevant to be tested on Cypriot students and it was of acceptable face validity.

### 3.5 Construct Validity

Construct validity was evaluated using exploratory factor analysis (EFA) with Varimax rotation. Principal components analysis (PCA) was implemented as the extraction method in EFA. The procedure used is similar to the psychometric studies published for the CLES+T (Saarikoski et al., 2008; Johansson et al., 2010; Henriksen et al., 2012; Bergjan & Hertel, 2013; Vizcaya-Moreno et al., 2015). First, the assumptions regarding the suitability of the data for factor analysis were examined, including the sample size, Kaiser–Mayer–Olkin (KMO) measure of sampling adequacy and Bartlett’s test of sphericity. The sample size was satisfactory, considering the rule-of-thumb for determining *a priori* sample size to be a “participant- to- item” ratio of 10:1 (Costello & Osborne, 2005). The current study, included 34 items and 463 participants, therefore this ratio was satisfied. The data were found to be appropriate for factor analysis, since the KMO measure was equal to 0.931, larger than 0.5, indicating high sampling adequacy, whereas Bartlett’s test of sphericity was significant (p<0.001), thus rejecting the null hypothesis of an identity correlation matrix (Field, 2009).

The criteria for factor selection included *eigenvalues* being higher than 1, as well as the percentage of variance explained by the factors, where each factor was expected to explain at least 5% of the variance to be included (Polit & Beck, 2011; Field, 2009). In the current study, the five-factor structure of the model that was obtained from EFA was identical to the original scale, where all the items loaded on each component agreed completely with the five sub-dimensions of the scale in Saariskoski et al. (2008). The five components had *eigenvalues* larger than one and explained a total of 67.4% of the variance. The most important factor for the learning environment of Cypriot nurses, which had the highest *eigenvalue* and the largest percentage of variance explained, was “supervisory relationship”, with an original *eigenvalue* of 13.1 (6.8 after Varimax rotation) and an explanation of around 38% of the variance (or 20% after rotation). The factor loadings in this component ranged from 0.78 up to 0.88. The loadings in all the other components were similarly very high and thus satisfactory, in that all were higher than 0.5. All the results (factor loadings of each item, eigenvalues and % of variance explained) appear in the Table 2 below:

**Table 2:** Factor loadings

Items on factor	Supervisory relationship (factor 1)	Role of nurse teacher (factor 2)	Pedagogical atmosphere (factor 3)	Premises of nursing in the ward (factor 4)	Leadership style of the Ward manager (factor 5)
My supervisor showed a positive attitude towards supervision	.844				
I felt that I received individual supervision	.783				
I continuously received feedback from supervisor	.826				
Overall I am satisfied with the supervision I received	.864				
The supervision was based on a relationship of equality and promoted my learning	.866				
There was a mutual interaction in the supervisory relationship	.864				
Mutual respect and approval prevailed in the supervisory relationship	.880				
The supervisory relationship was characterized by a sense of trust	.833				
The NT was capable of integrating theoretical knowledge and everyday practice of nursing		.812			
The NT was capable of operational sing the learning goals of this placement		.834			
The NT helped me to reduce the theory-practice cap		.805			
The NT was like a member of the nursing team		.757			
The NT was able to give his or her expertise to the clinical team		.824			
The NT and the clinical team worked in supporting my learning		.820			
The common meetings between myself mentor and NT were comfortable experience		.695			
In our common meetings I felt that we are colleagues		.698			
Focus on meetings was in my learning needs		.690			
The staff was easy to approach			.727		
I felt comfortable going to the ward at the start of my shift			.659		
During staff meetings(e.g. before shifts) I felt comfortable taking part in the discussions			.725		
There was a positive atmosphere on the ward			.755		
The staffs were generally interested in student supervision			.693		
The staff learned to know the students by their personal names			.609		
There were sufficient meaningful learning situations on the ward			.524		
The learning situations were multi-dimensional in terms of content			.516		
The ward can be regarded as a good learning environment			.508		
The ward nursing philosophy was clearly defined				.640	
Patients received individual nursing care				.741	
There were no problem in the information flow related to patients’ care				.691	
Documentation of nursing (e.g. nursing plans, daily recording of nursing procedures etc.) was clear				.684	
The WM regarded the staff on his/her ward as a key resource person					.685
The WM was a team member					.843
Feedback from the WM could easy be consider a learning situation					.794
The effort on individual employee was appreciated					.616

**Eigenvalues**	**13.065**	**3.750**	**3.179**	**1.654**	**1.270**
**% of variance explained (total=67.405%)**	**38.427**	**11.029**	**9.349**	**4.864**	**3.736**
**Eigenvalues (after rotation)**	**6.817**	**5.990**	**4.435**	**2.989**	**2.687**
**% of variance explained (total=67.405%) (after rotation)**	**20.049**	**17.618**	**13.045**	**8.791**	**7.902**

It should be noted that although item analysis did not show any significant deviations from normality, the principal axis factoring was implemented in EFA since it is a method without any distributional assumptions (e.g., Saarikoski et al., 2008; Johansson et al., 2010). However, the results from the two methods (principal components analysis and principal axis factoring) were identical in terms of the factor structure and grouping of items, although with slightly altered factor loadings, and therefore the results from the principal axis factoring are not reported.

In addition, it should be mentioned that, similar to Saarikoski et al. (2008), different models also were compared, namely with four and six factors. The 4-factor results were similar to the results reported by Saarikoski et al. (2008) where three dimensions were identical to the 5-factor solution and the fourth combined ward management and nursing care together, but in addition to the concern that one factor included too many items, the 4-factor model also explained a lower percentage of variance (63.7%). Finally, the 6-factor model, which was originally obtained, included an extra factor which included items 6-9 from the list of pedagogical atmosphere items, but the first two items (6 and 7), loaded similarly on the Pedagogical Atmosphere factor, with high loadings on both factors, therefore only the last three items (7-9), loaded more significantly on the sixth factor. Including this extra factor, with only these three items did not make sense intuitively. Therefore, the five-factor solution was the preferred and most appropriate model structure overall.

### 3.5 Convergent Validity

Convergent validity was examined by measuring the bivariate correlations between the scale/subscales and the question that measured the general satisfaction of nurses (replies to which optionally ranged from “fully dissatisfied” to “fully satisfied”). Spearman’s rho correlation coefficient was significant with p<0.001 between the overall satisfaction and all the scales, showing that the scale and subscales have convergent validity and indicating that all the subscales are important and need to be included in the scale. These results are presented in Table 3.

**Table 3:** Bivariate Correlations between the item on “total satisfaction” with all the scales/subscales.

Total satisfaction	Scale	Pedagogical Atmosphere	Ward Management	Nursing care	Supervisory relationship	Role of the Nurse Teacher
Spearman’s Correlation coefficient	0.610**	0.521**	0.388**	0.385**	0.550**	0.432**

p-value	<0.001	<0.001	<0.001	<0.001	<0.001	<0.001

** Correlation is significant at α=0.01.

### 3.6 Correlations Between the Subscales

Pearson correlation coefficient was used to examine the relation between the subscales. All the scales were highly significantly positively related, with p-values<0.001. These correlations are presented in Table 4.

**Table 4:** Correlations between the CLES-T subscales

		Pedagogical Atmosphere	Ward Management	Nursing care	Supervisory relationship	Role of the Nurse Teacher
Scale	PearsonCorrelation	0.794**	0.685**	0.708**	0.791**	0.748**
	p-value	p<0.001	p<0.001	p<0.001	p<0.001	p<0.001

PedagogicalAtmosphere	PearsonCorrelation		0.554**	0.577**	0.472**	0.402**
	p-value		p<0.001	p<0.001	p<0.001	p<0.001

WardManagement	PearsonCorrelation			0.537**	0.431**	0.381**
	p-value			p<0.001	p<0.001	p<0.001

Nursing care	PearsonCorrelation				0.459**	0.378**
	p-value				p<0.001	p<0.001

Supervisory relationship	PearsonCorrelation					0.460**
	p-value					p<0.001

** Correlation is significant at the 0.001 level.

## 4. Discussion

Examination of the psychometric properties of the CLES+T-GR demonstrated the internal consistency of the total scale, and its subscales as well, which was similar to results in other languages (Bergjan & Hertel, 2013; Henriksen et al., 2012; Johansson et al., 2010; Saarikoski et al., 2008; Vizcaya-Moreno et al., 2015). In the present study, three validation processes were applied: content, construct and convergent validity, indicating the degree to which scores measure what they claim to measure.

The results have shown that CLES+T-GR is a multidimensional instrument which consists of five factors, and is very similar to the first scale CLES-GR used without the “teacher-T” section (Papastavrou et al., 2010). In that study, the construct validity of the CLES-GR was examined using a sample of 350 students with an exploratory factor analysis. The total percentage of variance that the factor model explained was high (67%) and the questions loaded on the same factors as the factors in the original questionnaire. The reliability of the CLES-GR was satisfactory (Cronbach’s alpha =0.95) and the alpha values of the sub-dimensions ranged from 0.79 to 0.95.

The CLES+T is an extension of the CLES-GR that gives more possibilities to evaluate the new role dimensions of the nurse teacher in the clinical area. As nursing education has moved to the higher education systems so the CLES+T is thus more suited to the Cyprus Nursing educational system currently relied upon.

The factor model with a total explanation percentage of 67.4% of the variance found in our study was comparable with factor models of other studies in Europe, namely Germany 73% (Bergjan & Hertel, 2013) Spain 66.4% (Vizcaya-Moreno et al., 2015). Italy 67% (Tomietto et al., 2012) and Finland 64% (Saarikoski et al., 2008). The strongest factor was found in the subcategory “supervisory relationship” with high loadings ranging from 0.833 to 0.844, followed by the “role of the nurse teacher” factors with loadings ranging from 0.690 to 0.834. It is interesting that “supervisory relationship” has been identified as the strongest factor in most of the validated versions of the CLES+T (Saarikoski et al., 2008; Johansson et al., 2010; Henriksen et al., 2012; Bergjan & Hertel, 2013; Vizcaya-Moreno et al., 2015). Only in the Italian version (Tomietto et al., 2012) the strongest factor was the “pedagogical atmosphere” and “supervisory relationship” was the forth in sequence. Another interesting finding of our study is that “role of the nurse teacher” is the second strongest factor and as such is similar to the Norwegian sample (Henriksen et al., 2012). However, loadings were weaker than the Cypriot sample, ranging from 0.498 to 0.816, and also the question related to the nurse teacher cooperation loaded in another factor, that of the supervisory relationship. The least important factor of the clinical learning environment in our study was the “leadership style of the ward manager”, which in combination with the high importance given to the “role of the nurse teacher” in our study may be considered as shifting all of the learning responsibility to the nurse teacher, in turn minimizing the role of the ward manager. Initially the ward manager was recognized as a key person in the establishment of the ward as a good learning environment for student nurses in 1980’s (Orton, 1983), although subsequent studies found that the pedagogical activities of the clinical nursing staff are more important in the supervision of students (Mattila et al., 2010). Empirical evidence also gives more emphasis to models of “one to one” supervision rather than the traditional model of group supervision, and likewise to the role of the staff nurse in the clinical supervision process (Saarikoski et al., 2007). The strong support given to the role of the nurse teacher in our study also gives the message that students regard their teacher as more important in the process of learning and the person who will fulfill their expectations to reduce the theory-practice gap.

As mentioned, the CLES+T is validated in seven European countries and languages and despite the differences in examining the psychometric differences, the results show that in general almost all the items of the scale loaded on the same factor, although the robustness of each factor in each country varies. This is understandable in terms of the differences and complexities of health care and hospital organization systems that formulate the practice environment and influence student learning in each country. Also, although the cultural and social contexts of the learning environments in the countries mentioned is so varied, the advantage of CLES+T lies in the opportunity to use an instrument that measures all aspects of these diverse environments in a consistent way. This means that CLES+T is a strong, valid and reliable instrument that can be safely administered to all nurse students to evaluate their perceptions of various clinical settings as learning environments. Bachelor degrees in nurse education in Europe are characterized by different structures, standards and approaches to the relationship between theoretical and practice-based learning (Salminen et al., 2010). Hence, systematically processed empirical data collected with valid, reliable instruments are needed urgently for national and international comparisons, and to increase the pressures on policy makers.

### 4.1 Limitations

The limitations of the study include the issue of different study settings in that students were practicing in five different hospitals and many dissimilar wards. This means that the results may be different if analyzed at a hospital or unit level rather than nationally. Also, the comparisons made with validation studies from other countries need to be viewed with caution, as those studies did not implement any statistical comparative analyses of the factor loadings and so it is possible that certain results occurred by chance Finally, it would be interesting to employ other statistical methods of validation, as a confirmatory factor analysis.

## 5. Conclusions

The Greek version of the CLES+T has been shown to be reliable and valid. The CLES+T-GR evaluates the learning context of the clinical practice environment taking into consideration the multidimensional nature of the clinical area which involves aspects of the ward such as the pedagogical atmosphere, nursing care and ward management as well as supervision aspects and inter-staff relationships. The role of the teacher is central to the concept of the clinical learning environment, especially in integrating theoretical knowledge and everyday practice of nursing. CLES+T-GR enables researchers to use a methodological tool to assess the process of learning in applied science, such as nursing, that relies heavily on practice. Additionally, it facilitates the design of more effective, targeted interventions for improving the clinical learning environment. The results further suggest that there is still much to be done to promote improvements in the clinical area that will facilitate student learning and further research is needed into this area in the Greek context.
